# Making sense of the literature on antipsychotics and long-term functioning: taking natural history and personalization seriously

**DOI:** 10.1017/S003329172400312X

**Published:** 2024-12

**Authors:** Awais Aftab

**Affiliations:** Department of Psychiatry, Case Western Reserve University, Cleveland, OH, USA

**Keywords:** psychopharmacology, schizophreni, psychosis, natural history, iatrogenic, functioning, GAF, observational studies, discontinuation, dose reduction, personalization

## Abstract

This review examines the relationship between long-term antipsychotic use and individual functioning, emphasizing clinical implications and the need for personalized care. The initial impression that antipsychotic medications may worsen long-term outcomes is critically assessed, highlighting the confounding effects of illness trajectory and individual patient characteristics. Moving beyond a focus on methodological limitations, the discussion centers on how these findings can inform clinical practice, keeping in consideration that a subset of patients with psychotic disorders are on a trajectory of long-term remission and that for a subset of patient the adverse effects of antipsychotics outweigh potential benefits. Key studies such as the OPUS study, Chicago Follow-up study, Mesifos trial, and RADAR trial are analyzed. While antipsychotics demonstrate efficacy in short-term symptom management, their long-term effects on functioning are less obvious and require careful interpretation. Research on long-term antipsychotic use and individual functioning isn't sufficient to favor antipsychotic discontinuation or dose reduction below standard doses for most patients, but it is sufficient to highlight the necessity of personalization of clinical treatment and the appropriateness of dose reduction/discontinuation in a considerable subset of patients.

At first glance, the literature on the relationship between long-term antipsychotic use and individual functioning may suggest that antipsychotic medications worsen outcomes (Harrow & Jobe, 2018; Whitaker, 2011). However, this perceived association is deeply confounded by the natural course of psychotic disorders and by individual characteristics that influence the likelihood of continued medication use. The academic debate on this topic has been intense, and in communities critical of mainstream psychiatry – such as *Mad in America* and *Mad in the UK* – the belief that antipsychotics negatively impact functioning is widely accepted and promoted. In contrast, responses from the psychiatric community have often focused on highlighting the methodological flaws in these studies (Aftab, 2023; Leucht & Davis, 2017; Pierre, Zito, Yang, & Marder, 2023), leaving clinicians uncertain with regards to how these findings should inform clinical practice (with notable exceptions, such as Wunderink, 2019 and 2024).

This review aims to clarify the complicated literature on antipsychotic use and long-term functioning from the vantage point of clinical practice, considering the heterogeneity of natural course of psychotic disorders and its implications for personalized care. Rather than offering a comprehensive or systematic review of research studies, the focus of this paper will be on a few key studies that exemplify broader trends in the literature and will illustrate how they can be clinically interpreted with attention to the natural history of these disorders and the need for individualized treatment. I have limited the discussion to remission, recovery, and functioning, deliberately excluding related topics like changes in brain volume and the dopamine supersensitivity hypothesis which present distinctive methodological challenges with regards to interpretation. (My exclusion of these issues from the current discussion is not intended to imply that they are clinically irrelevant or that they have no bearing on decision-making by clinicians and patients.)

## Long term course and trajectories of psychotic disorders

To meaningfully interpret studies on long-term antipsychotic use, it is essential to first understand the natural course of psychotic disorders.

A recent 25-year longitudinal study by Tramazzo et al. (2024) from Suffolk County, USA, examined the illness trajectories of individuals following their first admission for psychosis. The study defined recovery based on both symptom severity and quality of life, while remission was defined by minimal or no symptoms over the past month. Among patients with schizophrenia spectrum disorders, approximately 25% experienced recovery or remission within the first four years. However, by the 25-year mark, these rates dropped sharply to 13% for recovery and 5% for remission. In contrast, individuals with other psychotic disorders (not schizophrenia spectrum) had remission and recovery rates above 50% for the first 20 years, which only fell below this threshold at the 25-year follow-up.

Interestingly, the study highlighted that recovery rates were generally higher than remission rates, indicating that many individuals can maintain good functioning despite persistent symptoms. Tramazzo et al., also found a high mortality rate of 20%, underscoring the long-term health challenges faced by those with psychotic disorders.

Although recovery rates in schizophrenia spectrum disorders are somewhat encouraging at 10 years (16%) and 20 years (22%), the rarity of sustained recovery and remission serves as a stark reminder of the chronic, recurrent nature of these conditions. The most common trajectory for individuals with schizophrenia spectrum disorders was no recovery or remission, with recovery or remission that is stable over two plus decades occurring in less than 1% of cases. In contrast, individuals with other psychotic disorders had much better outcomes, with stable recovery and remission rates of 21.1% and 15.1%, respectively.

The OPUS cohort study from Denmark mirrored the Suffolk findings, with only 14% meeting criteria for both symptomatic and psychosocial recovery at 10 years (Austin et al., 2013). This aligns with a comprehensive meta-analysis of recovery rates in schizophrenia (Jääskeläinen et al., 2013), which reported a median recovery rate of 13.5%, based on data from 50 studies.

A historical perspective further supports these findings. Taylor and Jauhar (2019) reviewed recovery rates over the past century, noting that even in the pre-antipsychotic era, around 15% to 20% of patients experienced prolonged periods of recovery, as seen in studies like those by Langfeld (1937) and Malamud and Render (1939). This historical comparison is instructive as it reminds us that the availability of treatments that acutely reduce symptoms and reduce the risk of relapse does not automatically translate into meaningful shifts in long-term recovery. Some possible and speculative explanations would include low rates of sustained treatment adherence, limited ability of current treatments to address neurocognitive deficits, relapses may be delayed but not entirely prevented, symptom suppression unaccompanied by etiological disease modification, and long-term benefits in some individuals being cancelled by iatrogenic harm in other individuals and contemporary availability of substances such as high-potency cannabis and stimulants.

An outlier to these findings is the AESOP study (UK), which reported a much higher rate of symptom remission. In a 10-year follow-up of 557 individuals with first-episode psychosis (219 successfully reinterviewed), 46% had been symptom-free for at least two years, including 40% of those with non-affective psychosis (Morgan et al., 2014). It is unclear what accounts for these differences; AESOP has a lower percentage of people with schizophrenia diagnosis, but even within the schizophrenia group, there is an under-representation of individuals experiencing a continuous course of psychosis and outcomes are better. The AESOP study, however, illustrates differentiation between symptom trajectories very well (see figure 2 in Morgan et al., 2014): Very few people with psychotic disorders in the AESOP cohort had a continuous course and a good outcome (2%). Vast majority of people with good outcome had an intermittent course.

We can roughly characterize the clinical trajectories of remission/recovery in psychotic disorders as follows:

1. Long-term remission/recovery after initial episode of psychosis
2. Long-term remission/recovery after multiple episodes of psychosis
3. Recurrent episodes of psychosis, with transient periods of remission/recovery
4. Chronic, persistent psychosis, without remission/recovery

In cross-sectional analyses of schizophrenia spectrum disorders, individuals in remission or recovery typically represent a mix of long-term recovered patients and those between episodes during a psychosis-free interval. Over time, those with long-term remission become overrepresented in the high-functioning group.

Understanding this is crucial because in long-term observational studies antipsychotic use overlaps with the natural course of psychotic disorders. While antipsychotics are known to reduce symptoms, lower the risk of relapse, and modestly improve functioning in the short term (up to about one year), there is no clear evidence that antipsychotics alter long-term trajectories of remission and recovery either way once we exclude those individuals who would have naturally recovered regardless of antipsychotic treatment.

Any long-term examination of antipsychotics in psychotic disorders must contend with the fact that the sample will display these different trajectories over time, and at present there is no clinical tool or measure that predicts these trajectories with accuracy. The people in the long-term recovery trajectory will naturally be the ones who will find it easy to discontinue antipsychotics and stay well.

## Efficacy and tolerability of antipsychotics in randomized controlled trials (RCTs)

Antipsychotics are generally effective in the short-term management of psychosis, improving not only symptom control but also quality of life and functioning. A comprehensive analysis by Leucht et al. (2017) of 167 RCTs conducted over 60 years reported a standardized mean difference (SMD) of 0.45 for positive symptoms (typically regarded as a medium-sized effect), with SMDs of 0.35 for quality of life and 0.34 for functioning (conventionally, small-sized effects), confirming moderate benefits in these domains.

For maintenance treatment, antipsychotics have shown similar efficacy, particularly in preventing relapse within the first year of treatment. A Cochrane review and meta-analysis by Ceraso et al. (2022) involving 75 RCTs found that antipsychotic medications significantly reduced relapse rates (24% *v.* 61% for placebo, 30 RCTs, RR = 0.38) and hospitalizations (7% *v.* 18% for placebo, 21 RCTs, RR = 0.43). These drugs also modestly improved quality of life (SMD = − 0.32, 95% CI =  −0.57 to −0.07) and social functioning (SMD = −0.43, 95% CI = −0.53 to −0.34).

However, these benefits come with a substantial risk of adverse effects, which can negatively impact functioning for some patients. Common side effects include sedation, akathisia, anticholinergic effects, and weight gain (Huhn et al., 2019). Ceraso et al. (2022) reported higher incidences of movement disorders (14% *v.* 8% for placebo), sedation (8% *v.* 5%), and weight gain (9% *v.* 6%) in maintenance studies.

The neuroleptic effect of chlorpromazine was initially described at the time of discovery as ‘sedation without narcosis.’ (Lewander, 1994) In addition to sedative effects such as drowsiness and somnolence, effects such as psychological indifference, apathy, psychological numbing, are also commonly experienced. We should expect these problems to be worse with high doses within the standard range, higher than standard doses, and with polypharmacy.

## Long-term considerations: discontinuation *v.* maintenance

A recent meta-analysis of 35 studies by Schlier et al. (2023) investigated how the effect of antipsychotic maintenance treatment *v.* discontinuation/dose-reduction on social functioning and subjective quality of life in patients with schizophrenia-spectrum disorders changes over the years.

They found that middle-term (2–5 years; 7 studies) and long-term follow-ups (>5 years; 2 studies) significantly favored discontinuation, but most of the middle- and all of long-term studies had a non-randomized study design and a high risk of bias, and therefore any conclusions are premature. Randomized studies in this meta-analysis were of higher quality and favored maintenance treatment but they were short-term (<2 years), while longer studies in this review were non-randomized, evaluated by authors as being of lower quality, and favored discontinuation. The most likely explanation for this is natural history, differences in psychological and developmental characteristics, and availability of psychological and social resources.

To better understand the methodological and interpretative challenges in this area, it is useful to explore three key studies from the Schlier et al. (2023) meta-analysis. These include the middle-term Mesifos trial., and the long-term OPUS and Chicago Follow-up studies, which are also among the most widely discussed in this field. In addition, I will review the findings of the recent RADAR trial, which explored dose reduction and discontinuation in patients with multi-episode schizophrenia spectrum disorders.

A common limitation of studies of long-term antipsychotics and functioning is that participants are generally on low doses of antipsychotics and polypharmacy is uncommon. Thus, the clinical subgroup that is potentially most likely to benefit from dose reduction in the real world is generally missing from these studies.

## OPUS cohort: 10-year outcomes (observational)

In a study by Wils et al. (2017), researchers followed 496 patients diagnosed with schizophrenia spectrum disorders as part of the Danish OPUS Trial. Ten years later, 61% of the original participants attended a follow-up assessment. 30% of these patients had achieved remission of psychotic symptoms and were no longer using antipsychotic medication.

The study population at the 10-year follow-up was divided into four groups. The remitted-off-medication, remitted-on-medication, and non-remitted-on-medication groups each comprised about 30% of the patients. The smallest group was the non-remitted-off-medication group, which included 10% of the patients.

Functioning, as measured by the Global Assessment of Functioning (GAF) scale, varied significantly between the groups. The ‘Remitted-off-medication’ group had the highest functioning (mean GAF score of 66.3), followed by the ‘Remitted-on-medication’ group (mean GAF 53.1). In the non-remitted groups, those not on medication also exhibited better functioning (mean GAF 48.1) compared to those on medication (mean GAF 42.4). Thus, in both remitted and non-remitted groups, individuals not on antipsychotics had higher functioning levels.

However, this study was cross-sectional, capturing data at the 10-year mark without examining the causal relationship between medication use and outcomes. As a result, it is subject to the same limitations related to natural disease progression, as discussed earlier. The authors are also careful not to make any causal assertions.

Those who successfully discontinue antipsychotics are in all likelihood systematically different from those who are unable to discontinue or unsuccessfully discontinue and then resume. They are likely to be either folks who are naturally on the long-term remission trajectory, or those with a recurrent course experiencing a transient periods of remission or those with chronic symptoms who have the psychological and social resources to manage symptoms without antipsychotic medications.

## Chicago follow-up study: 15- and 20-year outcomes (observational)

The Chicago Follow-up Study was designed to naturally observe and track the progress of individuals with serious mental illnesses over time. This long-term study focused on understanding the course of the illness, outcomes, symptoms, effects of medication, and recovery. The participants, who mostly had schizophrenia and affective psychosis, were followed for 20 years after their initial hospital admission. The first follow-up occurred two years after they left the hospital, with additional follow-ups at 4.5, 7.5, 10, 15, and 20 years post-discharge (Harrow & Jobe, 2007; Harrow, Jobe, & Tong, 2022).

Recovery was defined in a cross-sectional manner (over a 1-year period at the time of follow-up) and required absence of major symptoms throughout the follow-up year, adequate psychosocial functioning, and no psychiatric re-hospitalizations during the year.

For almost the entire 20-year period, those not on antipsychotics *at the time of a follow-up* had higher functioning compared to those on it. Those not on antipsychotics were also much more likely to be in a state of recovery compared to those on antipsychotics.

In contrast to the Wunderink, et al. (2013) study (see below), where the two groups had similar symptomatic remission at the 7-year mark (and differed on functional recovery), in the Harrow study, those on antipsychotics are also *more* psychotic compared to those not on antipsychotics: 64% on antipsychotic medications had psychotic activity compared to 28% not on psychiatric medications at the 15-year follow-up (see Figure 1; Harrow & Jobe, 2007). This is a major clue that what we are seeing in the Harrow study is natural history differentiation and not a causal effect of antipsychotic medications (in addition to the general difficulties with causal inference when it comes to naturalistic, observational design). Unfortunately, Harrow et al. (2022) are cavalier rather than cautious when it comes to presenting these outcomes as *effects of* antipsychotics.

In Harrow et al. (2022), 20% of schizophrenia patient in the study are characterized as never psychotic, 57% as intermittently psychotic, and 23% as always psychotic during the follow-up period. 24% were never prescribed antipsychotics during the study, 34% were intermittently prescribed, and 42% were always prescribed. The ‘never prescribed antipsychotics’ group is likely to be enriched with the ‘never psychotic’ group, and since the never psychotic group is naturally on a long-term remission with good outcomes trajectory, this biases the outcome associations we observe. There is also likely to be a strong overlap between the ‘intermittently psychotic’ and ‘intermittently prescribed antipsychotics’ group such that those prescribed antipsychotics at any given cross-sectional follow-up are those who are experiencing or those who have recently experienced an exacerbation of psychosis.

Much is made, by the authors and by some commentators, of the fact that even among those who were estimated to have a poor prognosis at baseline in the Harrow study, outcomes were worse for those on antipsychotics. This ignores the obvious explanation that even among those with chronic symptoms, those who successfully discontinue antipsychotics are systematically different from those who are unable to discontinue or those who discontinue and then have to resume. In the Harrow study, those not on antipsychotics at the 15-year follow-up had better prior functioning, had more internal locus of control, better self-esteem, better prognosis at baseline, and better premorbid developmental achievements (Harrow & Jobe, 2007). The only way to demonstrate otherwise is randomization or a rigorous control of confounding factors.

## Mesifos trial: 7-year outcomes (quasi-randomized)

In the Mesifos trial (Wunderink et al., 2007; Wunderink et al., 2013), researchers investigated the outcomes of treatment-naïve patients with first-episode psychosis (*n* = 128). At baseline, 45% of the participants were diagnosed with schizophrenia (likely an underestimate). After achieving six months of remission from positive psychotic symptoms, these patients were randomly assigned to either a dose reduction and discontinuation (DR) strategy or a maintenance treatment (MT) strategy.

At the two-year mark, relapse rate in the DR group was found to be twice as high compared to the MT group: 43% *v.* 21%. Additionally, patients in the DR group did not show improved functioning on average (Wunderink et al., 2007).

Following the initial two-year randomization period, patient care was managed at the discretion of their psychiatric clinicians. Five years later, a follow-up study was conducted looking at 7-year outcomes looking at 103 patients from the original cohort (Wunderink et al., 2013).

The seven-year follow-up revealed significant differences in outcomes. Patients originally assigned to the DR strategy had a higher rate of recovery (symptom remission and functional remission) compared to those in the MT group: 40.4% *v.* 17.6%. There were no apparent confounding factors influencing these results. Symptom remission rates (independent of functioning) were similar between the two groups, at 69.2% for the DR group and 66.7% for the MT group. Notably, relapse rates in the DR group eventually equaled those in the MT group by around the third year of follow-up, such over the 7-year period, the two groups had an overall equal number of relapses.

During the 2-year randomized phase, the mean daily dosages of haloperidol equivalents were 2.1 mg for the DR group and 2.9 mg for the MT group. The average difference is not that high at face value but was enough to produce a higher rate of relapse. The mean antipsychotic dose during the last two years of follow-up remained significantly different: 2.2 mg daily for the DR group *v.* 3.6 mg daily for the MT group.

During the two-year randomization phase, 21.5% of patients in the DR group successfully discontinued medication without relapse, 24.6% discontinued but had to restart due to relapse, and the rest reduced their dose without discontinuing. A few patients in the MT group also discontinued antipsychotics on their own.

At the seven-year follow-up, 21% from the DR group and 12% from the MT group had discontinued antipsychotics. Additionally, the same number of patients was using less than 1 mg of haloperidol equivalents daily during the last two years of follow-up. Thus, a total of 34 patients (33.0%) were on no or very low dose antipsychotic medication at the 7-year follow-up: 22 (42.3%) in the DR group and 12 (23.5%) in the MT group.

There are 3 ways to approach and interpret the Mesifos trial.

- Dose reduction/discontinuation during early years of treatment produces functional benefit years down the road.

If we want to strictly restrict causal inference to randomization, the Wunderink study is looking at the long-term functional effects of a dose reduction/discontinuation strategy only during the first 2 years of treatment of first-episode psychosis. In this scenario, what matters is what happened during the first 2 years of randomization, and what happened naturally during the subsequent 5 cannot be inferred as causally relevant. In other words, there is a functional benefit of early antipsychotic dose reduction but it doesn't become evident until years later.

1. It is impossible to establish a causal relationship between early dose reduction and better functional outcome at 7 years (and/or these are chance findings).

This is the view of critics such as Leucht and Davis (2017), and probably the view shared generally in the psychiatric community. It is true that ‘much can have happened in 5 years’ (Leucht & Davis, 2017) but it is also true that there is no other obvious explanation for the functional discrepancy other than early dose-reduction/discontinuation (Wunderink, 2019). This criticism is also related to the skeptical view that the findings may reflect type I error and, without pre-registration, our confidence in these findings should be low. The only way to really demonstrate that such a relationship exists is robust replication. If we can *repeatedly* demonstrate that dose-reduction/discontinuation shows better functional outcomes years later, we'd have to take that seriously.

1. Dose reduction/discontinuation produces better functional outcomes years down the road, but in order to see this difference, the DR group must continue to take antipsychotics at a dose lower than the MT group.

This possibility hasn't been discussed much in the literature, but it seems relevant because the DR group still had a lower average antipsychotic dose during years 5–7 compared to the MT group: 2.2 mg Haloperidol-equivalent daily for the DR group *v.* 3.6 mg daily for the MT group. Again, the only way to demonstrate whether this is the case would be robust replications of the Mesifos trial, such that in some replications the two groups are on similar antipsychotic dose during the years 5–7 years and in some replications, the two groups continue to differ. That would tell us whether only *early* dose-reduction matters or whether *persistent* dose-reduction is needed for improved functional outcomes.

## RADAR trial. 2-year outcomes (randomized)

The RADAR trial was specifically designed to compare the benefits and harms of a gradual process of antipsychotic reduction compared with maintenance treatment (Moncrieff et al., 2023). Unlike the Mesifos trial that was conducted in patients with first-episode psychosis, this one recruited people with multiple psychotic episodes or recurrent psychotic conditions. 69% of subjects in the study were diagnosed with schizophrenia. Researchers hypothesized that antipsychotic reduction would improve social functioning and that any increase in the rate of serious relapse (leading to psychiatric hospitalization) would be less than 10%. Antipsychotic medications were reduced gradually over a period of months in a flexible way, with the aim of discontinuing completely if possible (In most prior maintenance RCTs, discontinuation has been much more rapid, over days or a few weeks). 126 patients were assigned to dose reduction and 127 to maintenance treatment. 70% of participants in the reduction group reduced their antipsychotic dose by 50% or more.

Not only was there no difference between the maintenance and dose reduction groups, but social functioning also remained stable during the course of the trial over 24 months (Moncrieff et al., 2023). There was no worsening or improvement of social functioning with maintenance and no worsening or improvement with dose reduction. In contrast, the study found substantially higher rates of psychotic relapse in the dose-reduction group. By 24 months, 25% of the reduction group had at least one severe relapse, *v.* 13% of the maintenance group. 41% of the reduction group had a relapse of any severity *v.* 22% of the maintenance group.

The higher rate of relapse with dose reduction is consistent with the 2-year randomization phase of Mesifos (Wunderink et al., 2007) and it is also consistent with a meta-analysis (Højlund, Kemp, Haddad, Neill, & Correll, 2021) that showed that compared with standard doses of antipsychotics, low doses (50–100% of standard dose) increased the risk of relapse by 44% and very low dose (less than 50% of standard dose) increased the risk of relapse by 72%.

Participants allocated to antipsychotic dose reduction reached a median of 67% reduction of their baseline dose at some point during the trial. The median dose at 24 months was 33% less than at baseline (likely because those who relapsed on lower doses had their medication dose adjusted again to control symptoms). 34 (27%) of those randomized to reduction stopped their antipsychotic medication completely *at some time* during the 24-month follow-up period. By 24 months, only 13 people (10%) in the reduction group were not taking antipsychotics. That is, out of the 34 who stopped their antipsychotics completely at some point, 62% had to resume taking antipsychotics. This illustrates the difficulty posed by antipsychotic discontinuation in patients with schizophrenia spectrum disorders. Even in a trial where discontinuation was permitted and encouraged as a goal, only a fraction was able to stop their antipsychotics and stay off them. Successful dose reduction is often a more realistic outcome in the clinic than successful discontinuation (Steingard, 2018).

There was no difference in neurocognitive functioning (measured by a battery of tests that included digit span, digit symbol substitution, Rey auditory verbal learning, trail making, and verbal fluency) between the two groups. Dose reduction did not improve cognitive functioning in this group.

There were eight deaths in the reduction group during the study and four in the maintenance group.

It is important to keep in mind that the people in the study are a carefully selected group of people with relatively low risk. The study excluded those who lacked the capacity to consent to trial, those who had been required to take the medication under the Mental Health Act, those whom the clinicians considered to be at serious risk of harm to themselves or others, and people who had had a mental health crisis or hospital admission in the past month. For such patients, who constitute a considerable portion of patients in clinical practice, the risks of antipsychotic dose reduction or discontinuation are likely to be even higher.

Patients in this study had relatively mild symptom severity, were on reasonable doses of antipsychotics (around 8–10 mg of olanzapine or equivalent), and the side effect burden was mild, which is likely why no improvement in functioning or cognition was seen with dose reduction. The situation is likely to be different for folks who are on higher than standard doses of antipsychotics, who are receiving antipsychotic polypharmacy, or who are experiencing side effects while on standard doses of medications.

## Summary of methodological and interpretative issues

To the extent that we have been able to study the effects of antipsychotics under randomization, antipsychotics show beneficial effects on psychotic symptoms, relapse prevention, quality of life, and social functioning. Randomized trials, however, have generally been of less than 2-year duration. All studies longer than 2 years are either observational or quasi-randomized. With long-term observational studies of antipsychotics, we are forced to tackle the natural history of psychotic disorders and diverging clinical trajectories. We are also forced to confront the fact that those who successfully discontinue antipsychotics are systematically different from those who are unable to discontinue or unsuccessfully discontinue and then resume. They are likely to be either folks who are naturally on the long-term remission trajectory, or those with a recurrent course experiencing a transient periods of remission or those with chronic symptoms who have the psychological and social resources to manage symptoms without antipsychotic medications.

## Clinical implications

There are three situations in which it could be said that a person doesn't ‘need’ antipsychotics

- The person is in the long-term remission trajectory.
- The person can manage with their psychotic symptoms equally well (or reasonably well) via psychological and social interventions alone (this is a poorly characterized subset, but we know such patients exist).
- A third category would be the situation where antipsychotics are warranted but have proven to be ineffective or not tolerated (i.e. with adequate trials of multiple antipsychotics, including clozapine). Almost everyone is able to tolerate at least *some* of the available antipsychotics, but the ones tolerated might not be the ones effective.

Considering the effects of antipsychotics in light of these possibilities generates the following scenarios with regards to the net effect of antipsychotics on functioning:

**Scenario #1:** If a person stays on antipsychotics when they could've safely gotten off them (because they happen to be among those in a long-term remission trajectory or because they are in the poorly characterized subset of folks who can manage equally well via psychological and social interventions), the net effect on functioning is likely negative.

**Scenario #2:** If a person goes off antipsychotics that were effective for them, and they caused minimal side effects and the person tolerated them well, and if they have a recurrent or chronic form of psychotic illness, then they are at a higher risk of experiencing future episodes or exacerbations of psychosis, and their functioning is likely negatively affected by discontinuation (i.e. the net effect of antipsychotics on functioning is positive).

**Scenario #3:** If a person goes off antipsychotics that were effective for them and they caused substantial side effects, and if they have a recurrent or chronic form of psychotic illness, their functioning may improve or worsen or be unchanged depending on which – psychosis or medication adverse effects – had a bigger negative impact on their functioning.

**Scenario #4:** If a person goes off antipsychotics that were largely ineffective for them and they have a chronic persistent form of psychosis, their functioning may either stay the same (if they had minimal side effects) or may improve (if they had substantial side effects)

As reviewed in the initial section of this paper, there is considerable heterogeneity in reports from longitudinal studies. Scenario #1 probably applies to around 20–30% of people with first-episode psychosis (and to a substantially lower percentage of people with multi-episode schizophrenia spectrum psychosis) but this will vary from sample to sample (Chakraborty, 2021; Wunderink, 2024). Patients in this scenario possibly discontinue medications on their own, stay well, and don't stay in contact with clinical services. But many of them will be in clinical care, and clinicians have to actively consider dose-reduction and discontinuation as an option after a period of stability following psychosis, especially first-episode.

For people with recurrent psychosis who try dose reduction and discontinuation, they either find that they cannot successfully discontinue because psychotic symptoms soon return or they end up restarting antipsychotics for acute treatment at a later point when they experience psychosis again. Some people in scenario #3 may find it preferable to use antipsychotics only for acute treatment, but this strategy does come with accompanying risks, as many lose insight during acute states of psychosis, and there are serious legal, financial, and social risks to consider (incarceration, homelessness, alienating family, etc.) in addition to the disruption that hospitalization brings.

An important factor with regards to functioning and/or successful antipsychotic discontinuation is the availability of competent psychotherapists who are skilled in working with people with psychotic disorders. This is especially the case for those with recurrent or chronic symptoms. In my clinical experience, the people who successfully discontinue antipsychotics tend to be people with high premorbid functioning, who are highly motivated, engaged in psychotherapy, have good psychological insight, and strong social support.

If patients are actively experiencing medication effects such as feeling mentally dull, numb, ‘like a zombie,’ sedated, clinical experience suggests that a dose reduction or change in medication does usually improve functioning. It is also reasonable to think that those who experience metabolic adverse effects such as weight gain or pre-diabetes/diabetes can also experience decline in functioning, and dose reduction or medication switching can help with that. If a patient is tolerating a dose of the antipsychotic medication with minimal adverse effects and minimal impact on the patient's functioning (self-reported or otherwise), available literature doesn't provide strong evidence to suggest that dose reduction or discontinuation will be of functional benefit. The clinical goal, in my opinion, is to maintain patients on a medication dose, ideally near the low end of the therapeutic dose range, that they tolerate and that doesn't interfere with their functioning. Some, such as Wunderink, 2019, have proposed to gradually taper the antipsychotic dosage after first episode stabilization to half the daily defined dosage, ‘if increased relapse risks are acceptable.’ This is not unreasonable and will indeed benefit some people, but if a person is tolerating standard therapeutic dose without functional impairment, a higher risk of relapse in pursuit of an uncertain long-term functional benefit will not be acceptable to many patients and clinicians.

The problem arises when the medication or the dose of the medication required for clinical stabilization is one that actively produces adverse effects. E.g., when patients require high doses, or polypharmacy, or when they don't respond to first-line medications and require clozapine, which has more adverse effects and is less well tolerated. In such cases, we are forced to balance active symptom control or reducing the risk of relapse with active adverse effects. Ideally, the decision should be an informed one by the patient, but in practice, the culture when it comes to the treatment of schizophrenia spectrum disorders is extremely paternalistic. Clinicians want to minimize relapse, and other considerations are often secondary to them, even if those other considerations are more important to the patient. Resultantly, many patients are pressurized to stay on medication doses that they do not tolerate well, and patients are offered little to no support by the clinicians if they decide to reduce or discontinue the medication. Clinical decision-making here is made more complex when patients have poor insight or a poor appreciation of the risks, when there are concerns by family members about decompensation, and when, especially in places such as the US, there is a real risk of becoming homeless or being arrested. There are good reasons why clinicians who have skin in the game are so conservative in the management of psychotic disorders. While this clinical attitude of prioritizing relapse risk over functioning and tolerability keeps many patients stable, it also harms those who would've benefited from a personalized reduction, change, or discontinuation of medication.

An important consideration here is that there is a scarcity of established guidelines and practical ‘know-how’ when it comes to dose-reduction and discontinuation of antipsychotics (see Horowitz, Jauhar, Natesan, Murray, & Taylor, 2021, for a proposed method of tapering antipsychotics). For clinicians to be more responsive to patient preferences for dose reduction and discontinuation, the psychiatric community must address existing knowledge gaps with a research program that informs us about optimal tapering speed, accompanying psychosocial supports for successful tapering, and strategies for monitoring early warning signs.

All this demands personalization of antipsychotic treatment. This is rather obvious in many ways, and commentators on both sides of the debate are likely to agree on the need for personalization even when they disagree on what the ideal dose target is. Unfortunately, most clinical services continue to insist on long-term maintenance treatment for everyone and fail to support (or adequately guide) dose-reduction and discontinuation – driven by conservatism, paternalism, risk aversion, and outdated knowledge. On the other hand, in many critical and survivor spaces hostile to psychopharmacology, antipsychotics are demonized and everyone is encouraged to discontinue. Any current attempt at personalization of antipsychotic treatment will be crude and uncertain, but it is what patients deserve. Lex Wunderink puts it well: ‘Even small steps forward will easily outperform the current one-size-fits-all approach.’ (Wunderink, 2019)

## Conclusion

The clinician faces a difficult task when it comes to pharmacological treatment of psychosis. She has to be mindful of and take into account:

- Variable trajectory of psychotic disorders, with the most common trajectory in schizophrenia spectrum being no remission and no recovery, but a subset showing remission and recovery for extends periods of time.
- Antipsychotics are beneficial for the average patient in the acute treatment of psychosis and over 1–2 years, however, there are subsets of patient who do not respond adequately to antipsychotics (including clozapine) and for whom the adverse effects exceed benefits offered.
- Working with patients who lack insight into their condition, or who at high risk of suicide, violence, homelessness, or incarceration.
- A paternalistic medical and societal culture that prioritizes relapse prevention over patient functioning and quality of life.
- Long-term studies of antipsychotics are largely observational and do not adequately control for natural history or individual characteristics, while randomized trials of maintenance treatment show benefits with regards to symptom control, relapse prevention and functioning.
- A binary insistence on being on or off medications in communities critical of psychiatry, while ignoring dose reduction as a viable harm reduction strategy.

Psychiatric treatment of psychotic disorders has history suffered from a lack of recognition of natural history variation, and neglect of iatrogenic harm and patient preference driven by a culture of paternalism and genuine difficulties that arise in working with impaired insight, resulting in a lack of personalized treatment. The body of literature on the association of long-term antipsychotic use and functioning isn't sufficient to favor antipsychotic discontinuation or dose reduction below standard doses for most patients, but it is sufficient to highlight the necessity of personalization of clinical treatment.

## Funding statement

None.
